# Involvement of MicroRNAs in Probiotics-Induced Reduction of the Cecal Inflammation by *Salmonella* Typhimurium

**DOI:** 10.3389/fimmu.2017.00704

**Published:** 2017-06-13

**Authors:** Qiaoling Chen, Chao Tong, Shaoyang Ma, Luoxiong Zhou, Lili Zhao, Xin Zhao

**Affiliations:** ^1^College of Animal Science and Technology, Northwest A&F University, Yangling, China; ^2^Department of Animal Science, McGill University, Montreal, QC, Canada

**Keywords:** microRNAs, probiotics, *Salmonella*, ceca, chickens

## Abstract

The microRNAs (miRNAs) have been shown to play important roles in the development of the immune system and in regulation of host inflammation responses. Probiotics can effectively alleviate the inflammation caused by *Salmonella* in chickens. However, whether and how miRNAs are involved in modulation of the inflammation response in the gut of chickens have not been reported. In this study, the impact of a probiotics, *Lactobacillus plantarum* Z01 (LPZ01), was investigated on the cecal miRNAs and cytokine secretions in *Salmonella* Typhimurium (*S*. Typhimurium)-infected chickens at the age of 3 days. Newly hatched chicks were assigned to four groups (1): NC (basal diet) (2): S (basal diet + *S*. Typhimurium challenged) (3): SP (basal diet + *S*. Typhimurium challenged + LPZ01) (4): P (basal diet + LPZ01). In comparison with the S group, chicks in the SP group reduced the number of *S*. Typhimurium and had lower levels of interferon-γ and lipopolysaccharide-induced tumor necrosis factor alpha factor (LITAF) in ceca post challenge. Expression of 14 miRNAs was significantly affected by the presence of *S*. Typhimurium and/or *lactobacillus*. Five differential expression miRNAs (gga-miR-215-5p, gga-miR-3525, gga-miR-193a-5p, gga-miR-122-5p, and gga-miR-375) were randomly selected for confirmation by the RT-PCR. Predicted target genes of differentially expressed miRNAs were enriched in regulation of cAMP-dependent protein kinase activity, stress-activated MAPK cascade, immune system development and regulation of immune system process as well as in immune related pathways such as MAPK and Wnt signaling pathways. The relationship between changes of miRNAs and changes of cytokines was explored. Finally, 119 novel miRNAs were identified in 36 libraries totally. Identification of novel miRNAs significantly expanded the repertoire of chicken miRNAs and provided the basis for understanding the function of miRNAs in the host. Our results suggest that the probiotics reduce the inflammation of the *S*. Typhimurium infection in neonatal broiler chicks, at least partially, through regulation of miRNAs expression.

## Introduction

Salmonellosis is one of the most common and widely distributed foodborne diseases with tens of millions of human cases worldwide every year. One of the main causes for Salmonellosis in humans is contaminated poultry-derived foods, mainly eggs and egg products but also chicken meat. *Salmonella* Typhimurium (*S*. Typhimurium) infect many animal species, including humans and poultry, resulting in a spectrum of outcomes ranging from severe disease to asymptomatic carriage. Salmonellosis in the human is mainly caused by *Salmonella* Enteritidis (*S*. Enteritidis) and *S*. Typhimurium (1). *S*. Typhimurium infection in chicks younger than 2 weeks old also results in enteric and systemic disease with a high mortality rate, while infection in older chicks results in asymptomatic cecal colonization with persistent shedding of the organisms in feces (2).

Decreasing the prevalence of *Salmonella* in poultry flocks may effectively reduce salmonellosis in humans. For example, in Belgium, as in most of the EU, the incidence of salmonellosis has decreased since 2003 after the implementation of national plans (3). Many effective management strategies to control *Salmonella* infection in poultry include preventive hygienic measures, nutritional, and immune modulation strategies (3). Among the immune modulation strategies, probiotics confer the health benefit on the host when administered in adequate amounts. In addition to be used for control of specific enteric pathogens, probiotics are becoming accepted as potential alternatives to antibiotics as growth promoters. The probiotics can effectively reduce the *Salmonella* colonization, possibly through the competitive exclusion by probiotic bacteria. Despite the fact that many studies have showed the beneficial effects of probiotics for reducing *Salmonella* infection in chickens, the underlying mechanisms for the health benefits of probiotics, specifically for reduction of *Salmonella* infection, are still not fully understood.

Mature microRNAs (miRNAs) are an abundant class of small non-coding RNAs with 18–25 nt in length that posttranscriptionally modulate the expression of their target genes by binding to the 3′-untranslated regions (4). They participate in development of the immune system and in regulation of host inflammation responses to the gut microbes, including both pathogenic and non-pathogenic bacteria (5). During comprehensive analyses of the impact of two *Lactobacillus* species, *Lactobacillus paracasei* CNCM I-3689 and *Lactobacillus casei* BL23, on *Listeria monocytogenes* infection in a gnotobiotic mouse model, Archambaud et al. (6) found that three miRNAs (miR-192, miR-200b, and miR-215) were repressed during *L. monocytogenes* infection and treatment with each Lactobacillus increased miR-192 expression, whereas only *L. casei* increased miR-200b and miR-215 expression in ilea. The situation is different in conventional mice due to the presence of the endogenous microbiota. *L. monocytogenes* infection reduced expression of miR-143, miR-148a, miR-200b, miR-200c, and miR-378 in intestine upon *L. monocytogenes* infection (7). Similarly, Singh et al. (8) demonstrated that 16 miRNAs were differentially expressed in ceca of germ-free versus conventional mice. Nata et al. (9) reported that overexpression of miR-146b activated the NF-κB pathway, improved epithelial barrier function, ameliorated intestinal inflammation in dextran sulfate sodium-induced colitis mice. Hoeke et al. (10) found that *Salmonella* infection led to miR-29a upregulation by targeting caveolin 2 in the ileum of piglets. These studies exemplified changes and functions of miRNAs in the intestine of different animals reacting to normal microbiota or pathogenic bacteria. However, up to now, there is little information for miRNAs in poultry intestine.

In this study, we hypothesized that *Salmonella* infection and probiotic supplementation might change miRNAs expression in ceca of neonatal broilers. In order to evaluate whether supplementation of probiotics was effective to change miRNAs expression and thus reduce *Salmonella* infection in neonatal broilers, we performed the miRNA sequencing of cecal samples from *Salmonella* infected and probiotic-supplemented chicks in addition to measuring the mRNAs expression of three pro-inflammatory cytokines and loads of *Salmonella* in the ceca.

## Bacterial Culture

A *S*. Typhimurium (CVCC542) strain with novobiocin resistance was obtained from the China Veterinary Culture Collection Center and used in this study. The bacterium was cultured at 37°C in the Luria Bertani (LB) medium (Difco, Franklin Lakes, NJ, USA) containing 25 µg of novobiocin per milliliter for 16 h with shaking before being used for the challenge. The number of colony-forming units (CFUs) of *S*. Typhimurium was determined by plating serial dilutions on plate count agar plates and 1 × 10^8^ CFU in 0.2 mL sterilized phosphate-buffered saline (PBS, pH 7.2) was used to infect chickens.

## Probiotic Culture

The *Lactobacillus plantarum* Z01 (LPZ01), which was previously used in the study of Feng et al. (11), was grown on the Man-Rogosa-Sharpe Broth (MRS, Difco) under anaerobic conditions at 37°C for 24 h every time before use. Whole cells of the LPZ01 were obtained by centrifugation at 12,000 × *g* for 10 min at 4°C and washed twice with PBS. Then the LPZ01 culture was diluted in reconstituted powdered skim milk to an expected concentration of 1 × 10^8^ CFU/mL for oral gavage to chicks. Actual CFUs administered per chick were determined retrospectively.

## Birds, Experimental Design, and Sample Collection

**Figure 1:**
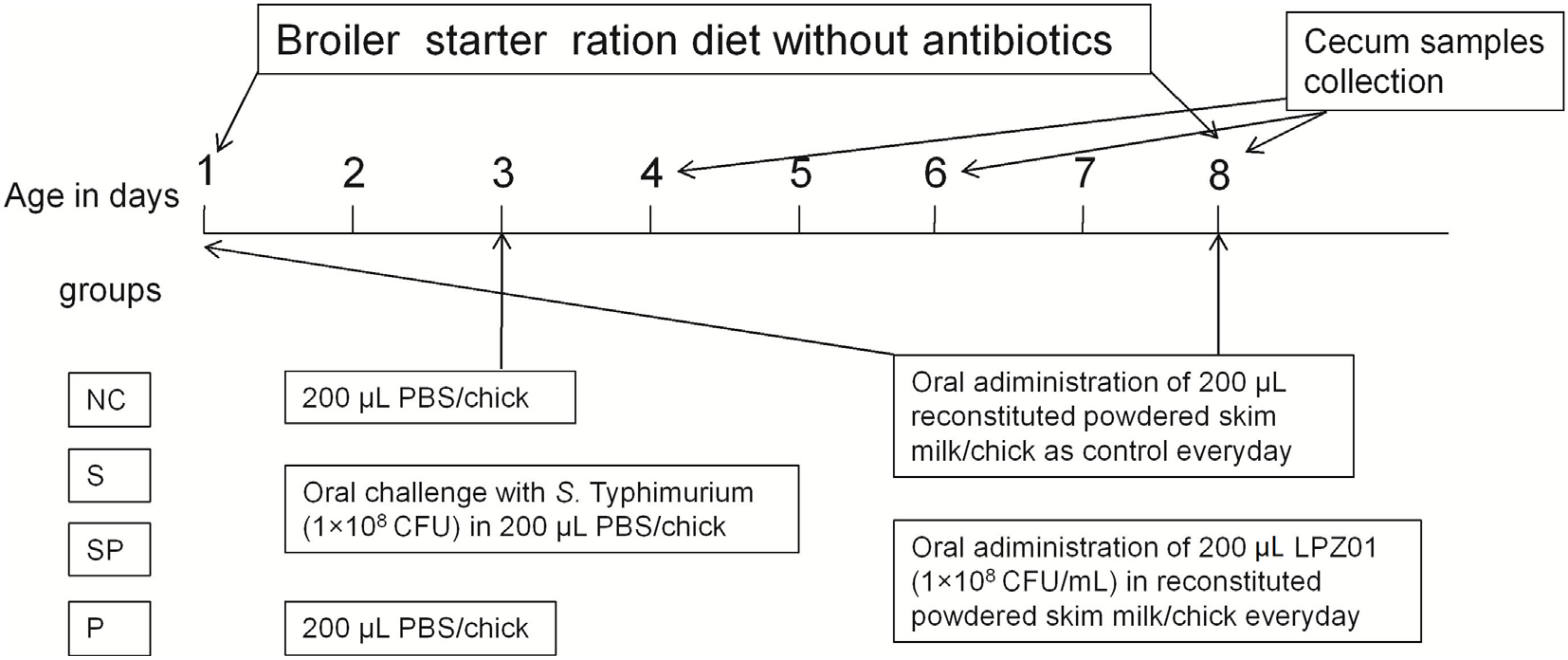
The experimental design. Newly hatched chicks were divided into four treatment groups (*n* = 6 cages/treatment; 6 chicks in each cage), (1) an uninfected negative control group (NC: negative control); (2) a positive control group infected with *S*. Typhimurium (S: *S*. Typhimurium); (3) a group infected with *S*. Typhimurium and orally supplemented with *Lactobacillus plantarum* Z01 (LPZ01) (SP: *S*. Typhimurium + probiotic); (4) a group only orally supplemented with LPZ01 (P: probiotic).

Newly hatched commercial male broiler chickens (Arbor Acres) were obtained from a local hatchery in Xianyang city, Shaanxi province of China. Birds were housed and maintained in a clean and sterile house with age-appropriate temperatures and 60–70% humidity, receiving water and diets *ad libitum*. A standard chick starter diet, containing no antimicrobial additives, was formulated according to the NRC requirement (23% protein and 3,029 kcal/kg metabolizable energy) and the main ingredients of the diet were corn and soybean meal. A total of 144 chicks were randomly assigned into four treatments, including (1) an uninfected control group (NC: negative control); (2) a positive control group infected with *S*. Typhimurium (S: *S*. Typhimurium); (3) a group infected with *S*. Typhimurium and orally supplemented with LPZ01 (SP: *S*. Typhimurium + probiotic); (4) a group only orally supplemented with LPZ01 (P: probiotic) (Figure 1). Each treatment had six cages with six birds in each cage. As shown in Figure 1, infections were carried out by oral gavage of 0.2 mL novobiocin-resistant *S*. Typhimurium (1 × 10^8^ CFU in 0.2 mL) at 3 days of age in the S and SP group, whereas chicks in the NC and P groups orally received 0.2 mL PBS. Birds were moved to another house before challenge. LPZ01 in reconstituted powdered skim milk was administered *via* oral gavage (0.2 mL) to SP and P groups every day, while other two groups received reconstituted powdered skim milk only.

Six birds per treatment were euthanized by cervical dislocation on days 1, 3, and 5 after the *S*. Typhimurium challenge. From each bird, one of the ceca (with contents) was placed in a 2 mL tube containing 1 mL of sterile PBS, weighted, and grinded with Scientz-48 Tissue Grind (Scientz Biotechnology Co. Ltd., Ningbo, China) for *Salmonella* enumeration as previously described (12). Another cecum was collected from each bird, placed in the RNA later RNA Stabilization Reagent (QIAGEN, Shanghai, China) and stored at −80°C until RNA extraction. All experimental protocols used in this experiment were approved by the Northwest A&F University Institutional Animal Care and Use Committee (protocol number NWAFAC1025).

## Detection of Cecal *Salmonella*

Homogenized cecal samples (with contents) were serially diluted in sterile PBS right after sample collection. From each dilution, 0.1 mL was then plated in triplicate on Brilliant Green Sulfa Agar (BD Diagnostics, Sparks, MD, USA) containing 25 µg of novobiocin/mL. Plates were incubated for at least 24 h at 37°C. The average values of number of *Salmonella* CFU were used for the statistical analysis. *Salmonella* counts between the S and SP groups were compared using the Student’s *t*-test using the SPSS for Windows version 17.0 (Chicago, IL, USA). The *P* < 0.05 was considered statistically significant.

## Small RNA Library Construction and Solexa Sequencing

Thirty six miRNA libraries from four treatments at three time points with three repeats in each treatment were constructed. Small RNAs were extracted with the mirVana™ miRNA Isolation Kit (Cat#. AM1561, Ambion, Austin, TX, USA) according to the manufacturer’s instruction. RNA integrity and concentration were confirmed using an Agilent 2100 Bioanalyzer (Agilent technologies Santa Clara, USA). Low molecular weight RNAs were separated by 15% polyacrylamide gel electrophoresis (PAGE) and RNA molecules in the range of 18–35 nt were enriched and ligated with proprietary adapters to the 5′ and 3′ terminal. A reverse transcription reaction followed by PCR was performed to obtain sufficient products for Illumina deep sequencing with HiSeq 2500 (Illumina) by the Illumina Genome Analyzer in Shanghai Biotechnology Corporation (Shanghai, China).

## Bioinformatics Analysis of Deep Sequencing Data

The raw sequences were preprocessed by the FASTXToolkit (fastx_toolkit-0.0.13.2^1^) to filter out the low-quality reads, as well as sequences with 3′ primers, with 5′ primer, with polyA tails, or with insert tags. Further annotation analyses were performed using the commercial software CLC Genomic Workbench 5.5. Briefly, the resulting clean reads were aligned against various databases, including ncRNA, piRNA, and Rfam, allowing a maximum mismatch of 2 nt to remove non-coding RNA, such as rRNA, tRNA, snRNA, and snoRNA. The remaining reads were analyzed by BLAST searches against chicken miRNA mature sequences and precursor structures that have been annotated in the miRBase (version 21.0) to identify conserved miRNAs. Only those small RNAs perfectly matching known chicken miRNAs or precursors in the miRBase were considered as conserved mature miRNAs. Sequences that cannot match the sequences in the above database were analyzed by the miRCat program in the sRNA Toolkitsoft page^2^ (13) against the chicken genome. The genomic hit distribution patterns and secondary structure of genomic regions corresponding to sRNA hits were observed and mature miRNAs and their precursor structures were predicted. The precursors with stem-loop hairpins were predicted when their minimum free energy and Randfold *P*-value were lower than −20 kcal/mol and 0.1, respectively (14). After all annotation steps, the sequences were used for size distribution and saturation analyses.

## Analyses of Differentially Expressed miRNA

To compare differentially expressed miRNAs among multiple samples, the sequences less than five total reads counts were removed first in the all libraries and the miRNA abundance of the remaining reads was normalized as RPKM (reads per kilobase per million reads) and the edgeR package (15) was used to identify statistically differential expression miRNAs. Differentially expressed miRNAs were defined as *P*-value <0.05 and |logFC| > 1. The heatmap of these differentially expressed miRNAs was constructed using the HemI software (16).

## Prediction of Differentially Expressed miRNA Targets and Gene Ontology (GO) and Kyoto Encyclopedia of Genes and Genomes (KEGG) Pathway Analyses

Based on the sequences of the miRNAs, the target genes of differentially expressed miRNAs were predicted using the online database miRDB^3^ (17) and TargetScan. The common target genes predicted by both software programs were selected for GO functional annotation and KEGG pathway analyses, unless otherwise stated. The KOBAS (kobas3.0-20170316) (18) software was used for GO and KEGG pathway analyses. The Fisher’s exact test was used and a *P*-value less than 0.05 was used as the cutoff criterion for the GO and KEGG pathway analyses. The KEGG pathway plot was produced by the ggplot2 package (19).

## Validation of Differentially Expressed miRNAs and Cytokine Expression in Cecal Samples

Five randomly selected differentially expressed miRNAs were validated by the stem-loop RT-PCR (20) and relative expression of cytokine genes in the cecal tissue was determined by RT-PCR. Total RNA was extracted from every cecal sample using the RNAiso Plus (Takara, Dalian, China) according to the manufacturer’s instruction. The RNA quantity was checked using a NanoDrop 2000 spectrophotometer (Thermo Scientific, MA, USA) and RNA was then converted to cDNA with the miRNAs RT special primer mixture or oligo (dT) primers, using the PrimeScript RT reagent Kit with gDNA Eraser (Takara). The cDNA was used as a PCR template for real-time qPCR quantification of miRNA or cytokine mRNA expression. The chicken 5s rRNA gene and GAPDH gene were used as endogenous controls of miRNAs and cytokines genes, respectively. The qPCR was performed using a Bio-Rad CFX 96™ Real-Time Detection System (Bio-Rad Laboratories, USA) and SYBR Green PCR Master Mix (Takara) in a final volume of 20 µL. The primers for miRNAs and cytokine genes were designed and listed in Table 1. All reactions were carried out in triplicate. The thermal cycle profile included an initial activation step at 95°C for 30 s, followed by 40 cycles of 95°C for 10 s (denaturation), 55–60°C (depending on the primer as described in Table 1) for 20 s (annealing) and 72°C for 10 s (extension). The data were the means of three separate experiments and were analyzed using the 2^−ΔΔCT^ method (21).

**Table 1:** Primers used for qPCR analyses.

Target	Primer	Sequence (5′–3′)	Annealing Temp (°C)	Products (bp)	Reference
IL-6	F^a^ R^b^	ATCCCTCCTCGCCAATCT GGCACTGAAACTCCTGGTCT	58	142	(12)
Interferon-γ	F R	ATCATACTGAGCCAGATTGTTTC CGCCATCAGGAAGGTTGT	56	124	(12)
Lipopolysaccharide-induced tumor necrosis factor alpha factor	F R	TACCCTGTCCCACAACCTG TGAACTGGGCGGTCATAGA	58	152	(12)
GAPDH	F R	TGGAGAAACCAGCCAAGTAT GCATCAAAGGTGGAGGAAT	55	145	(12)
Gga-miR-215-5p	RT^c^ F	GTCGTATCCAGTGCAGGGTCCGAGGTATTCGCACTGGATACGACGTCTGT GCCGCATGACCTATGAATTG	60	60	This study
Gga-miR-3525	RT F	GTCGTATCCAGTGCAGGGTCCGAGGTATTCGCACTGGATACGACTCACAGA GCCCGCAGCCATTCTGCGAT	60	61	This study
Gga-miR-193a-5p	RT F	GTCGTATCCAGTGCAGGGTCCGAGG TATTCGCACTGGATACGACTCATCTC GCCCGTGGGTCTTTGCGGGC	60	61	This study
Gga-miR-122-5p	RT F	GTCGTATCCAGTGCAGGGTCCGAGG TATTCGCACTGGATACGACACAAAC GCCCGTGGAGTGTGACAATGGT	60	62	This study
Gga-miR-375	RT F	GTCGTATCCAGTGCAGGGTCCGAGG TATTCGCACTGGATACGACTAACGC GCCCGTTTGTTCGTTCGGCTC	60	61	This study
Universal Primer^d^	R	GTGCAGGGTCCGAGGT	60	–	This study
5s rRNA	F R	GGAGGTCTCCCATCCAAGT AACGCCCGATCTCGTCT	60	97	This study

*^a–c^F, R, and RT represents the forward primers, reverse primers and RT primers, respectively*.

*^d^The universal primer is reverse primers for all the miRNAs*.

## Results

### Effect of LPZ01 on the Cecal Number of *Salmonella*

No *S*. Typhimurium colony was recovered from the ceca of the uninfected groups. In comparison with the S group, LPZ01 significantly reduced (*P* < 0.05) the *S*. Typhimurium counts by 97.92, 83.37, and 42.37% at day 1, day 3, and day 5 after the *Salmonella* challenge, respectively (Table 2).

**Table 2:** The effects of *Lactobacillus plantarum* Z01 on the *Salmonella* counts from the cecal content of chicks after 1, 3, and 5 days postinoculation with *Salmonella* Typhimurium.

Cecal content colony-forming unit (×10^5^)/g	1 day postinfection	3 days postinfection	5 days postinfection
S	252 ± 12.65^a^	44.01 ± 3.28^a^	17.82 ± 4.31^a^
SP	5.24 ± 0.59^b^	7.32 ± 1.06^b^	10.27 ± 1.16^b^

*^a,b^Different superscripts indicate significant differences between two treatments on each sampling day (*n* = 6) (*P* < 0.05)*.

### Analyses of Sequencing Data

More than 10,000,000 clean raw reads were generated from each of 36 samples. After trimming of adaptors and eliminating the low-quality reads, more than 9,000,000 effective reads were obtained for each sample. The effective read ratio of the samples was more than 90% and most of them were more than 95% (Table 3). The majority of reads for small RNAs were 20–24 nt in length and the highest numbers of reads appeared at the length of 22 nt (Figure S1 in Supplementary Material).

**Table 3:** The effective reads in each cecal sample.

Sample names	Raw reads	Effective reads	Effective ratio (%)
NC-1-d1	11674094	11362318	97.33
NC-2-d1	11927955	11562006	96.93
NC-3-d1	14088314	13687710	97.16
S-1-d1	14011120	13611121	97.15
S-2-d1	14194389	13594980	95.78
S-3-d1	13581455	13215944	97.31
SP-1-d1	15205829	14847623	97.64
SP-2-d1	12717398	11793339	92.73
SP-3-d1	11348514	10962190	96.60
P-1-d1	11824330	11363566	96.10
P-2-d1	14346747	13970857	97.38
P-3-d1	11212111	10815805	96.47
NC-1-d3	12755393	12285995	96.32
NC-2-d3	10907617	10537922	96.61
NC-3-d3	10663781	10284334	96.44
S-1-d3	11043881	10439583	94.53
S-2-d3	11140241	10603266	95.18
S-3-d3	12833702	12547738	97.77
SP-1-d3	11832194	11583722	97.90
SP-2-d3	10003511	9292114	92.89
SP-3-d3	10365290	9881859	95.34
P-1-d3	10898761	10392383	95.35
P-2-d3	12139605	11714304	96.50
P-3-d3	11086404	10430217	94.08
NC-1-d5	12422320	11448626	92.16
NC-2-d5	10513113	9521587	90.57
NC-3-d5	10797484	9975452	92.39
S-1-d5	11251604	10500226	93.32
S-2-d5	12021504	11678259	97.14
S-3-d5	11126963	10879395	97.78
SP-1-d5	10709176	10399217	97.11
SP-2-d5	11505173	10484676	91.13
SP-3-d5	15151240	14177115	93.57
P-1-d5	11639001	11376428	97.74
P-2-d5	14720414	14089745	95.72
P-3-d5	13765187	13082632	95.04

In mature known chicken miRNAs of the 36 libraries, 20 highly expressed miRNAs accounted for 89.16% of the total reads of identified known miRNAs (Table 4). The top five highly expressed miRNAs were gga-miR-215-5p, gga-miR-10b-5p, gga-miR-21-5p, gga-miR-26-5p, and gga-miR-22-3p, accounting for 40.54, 7.76, 6.78, 6.43, and 6.35% of total known miRNA reads, respectively.

**Table 4:** Top 20 expressed microRNAs (miRNAs) in chicken ceca in all treatment groups.

miRNA name	Total number of reads	Ratio^a^				
		All samples (%)	NC group (%)	S group (%)	SP group (%)	P group (%)
miR-215-5p	68917434	40.54	43.57	42.47	33.68	42.12
miR-10b-5p	13186219	7.76	7.56	7.24	9.67	6.76
miR-21-5p	11519323	6.78	5.75	6.43	8.09	6.80
miR-26a-5p	10931445	6.43	6.36	6.43	6.91	6.08
miR-22-3p	10803319	6.35	5.80	6.10	7.02	6.47
miR-10a-5p	4743039	2.79	2.65	2.63	3.23	2.67
miR-148a-3p	4079948	2.40	2.26	2.36	2.72	2.28
miR-194	3999556	2.35	2.55	2.52	1.94	2.39
miR-92-3p	3254438	1.91	1.92	1.88	1.94	1.91
miR-30d	3206318	1.89	1.8	1.78	2.02	1.94
miR-181a-5p	3106586	1.83	1.79	1.85	1.98	1.71
miR-429-3p	2497698	1.47	1.38	1.39	1.54	1.55
let-7f-5p	2310212	1.36	1.33	1.31	1.49	1.31
miR-30a-5p	1651325	0.97	0.93	0.97	1.05	0.94
miR-133a-3p	1586620	0.93	0.90	0.94	1.02	0.89
miR-199-3p	1430584	0.84	0.81	0.82	0.95	0.80
miR-30c-5p	1419796	0.84	0.84	0.77	0.84	0.88
miR-200a-3p	1229985	0.72	0.69	0.68	0.77	0.75
miR-126-5p	1190078	0.70	0.67	0.69	0.77	0.67
miR-27b-3p	1075489	0.63	0.52	0.61	0.75	0.64
Total for the above	152139412	89.16	90.08	89.87	88.38	89.57

*^a^The ratio refers to the total number of reads of a miRNA as compared to all detected reads of known chicken miRNAs (miRBase Release 21.0)*.

One hundred 19 novel miRNAs were identified from the candidate miRNAs that met both the expression and biogenesis criteria. The length of novel miRNAs ranged from 18 to 23 nt and the sequences of the mature miRNA and miRNA precursors of these novel ones were identified and shown in Table S1 in Supplementary Material.

### Validation of Differentially Expressed miRNAs

**Figure 2:**
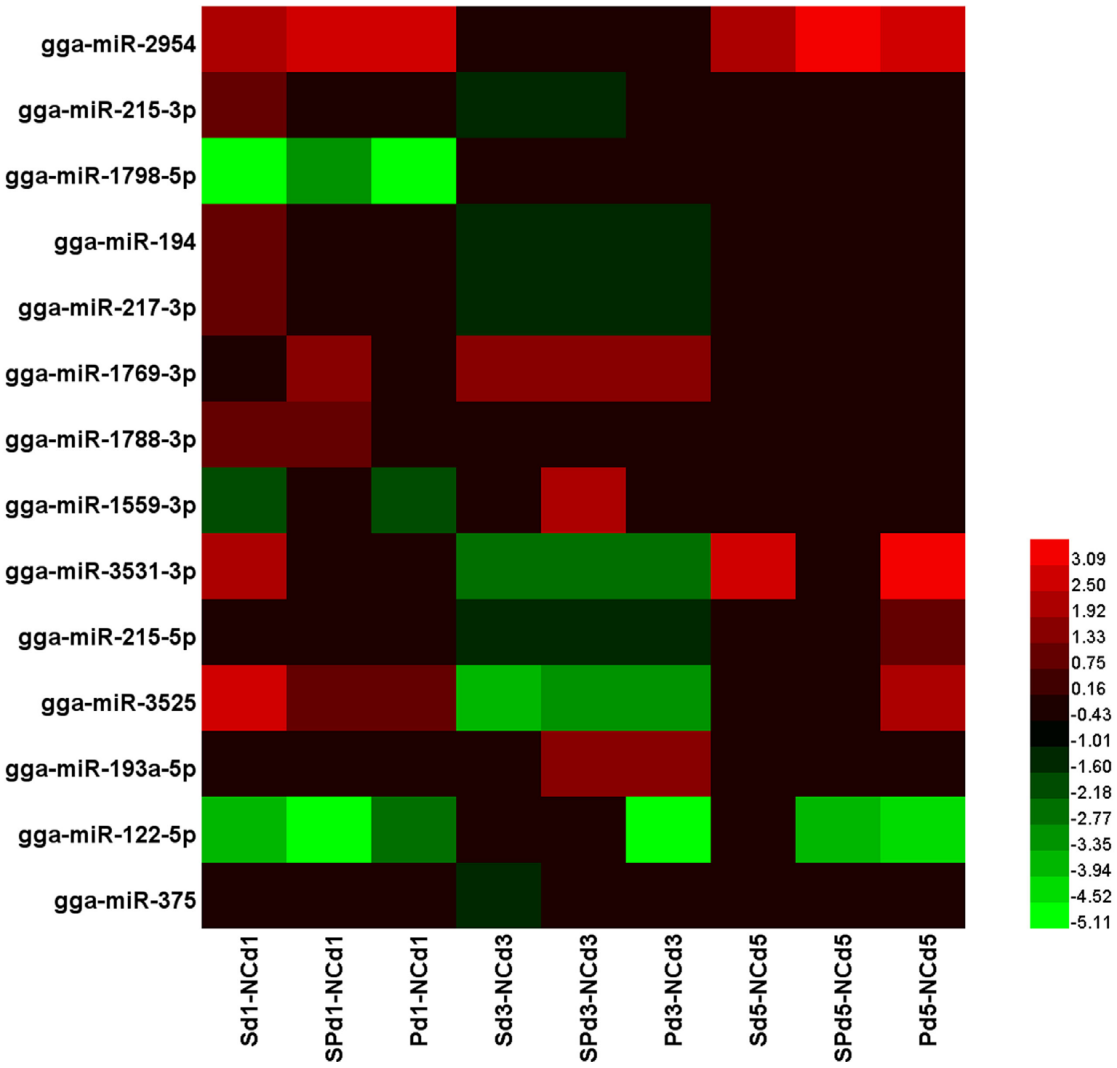
The heatmap of 14 differentially expressed microRNA (miRNAs) (*P* < 0.05, |logFC| > 1) in the chicken ceca, with or without *Salmonella* Typhimurium infection as determined by edgeR analysis of normalized sequence reads corresponding to known chicken miRNAs, logFC was computed as log_2_ between the treatment groups and the NC group. The heatmap for each miRNA was calculated based on its minimal and maximal recorded expressions. Sd1-NCd1: the difference between the S group and the NC group 1 day after *S*. Typhimurium infection; SPd1-NCd1: the difference between the SP group and the NC group 1 day after *S*. Typhimurium infection; Pd1-NCd1: the difference between the P group and the NC group 1 day after *S*. Typhimurium infection; Sd3-NCd3: the difference between the S group and the NC group 3 days after *S*. Typhimurium infection; SPd3-NCd3: the difference between the SP group and the NC group 3 days after *S*. Typhimurium infection; Pd3-NCd3: the difference between the P group and the NC group 3 days after *S*. Typhimurium infection; Sd5-NCd5: the difference between the S group and the NC group 5 days after *S*. Typhimurium infection; SPd5-NCd5: the difference between the SP group and the NC group 5 days after *S*. Typhimurium infection; Pd5-NCd5: the difference between the P group and the NC group 5 days after *S*. Typhimurium infection.

To determine the effects of *S*. Typhimurium and LPZ01 on miRNA expression in chicken ceca, expression of miRNAs in groups PC, S, and SP was compared with the NC group at three sampling time points. A total of 14 differentially expressed miRNAs were detected (gga-miR-2954, gga-miR-215-3p, gga-miR-1798-5p, gga-miR-194, gga-miR-217-3p, gga-miR-1769-3p, gga-miR-1788-3p, gga-miR-1559-3p, gga-miR-3531-3p, gga-miR-215-5p, gga-miR-3525, gga-miR-193a-5p, gga-miR-122-5p, and gga-miR-375). The heatmap analysis showed the relative changes of these miRNAs relative to the NC group (Figure 2).

**Figure 3:**
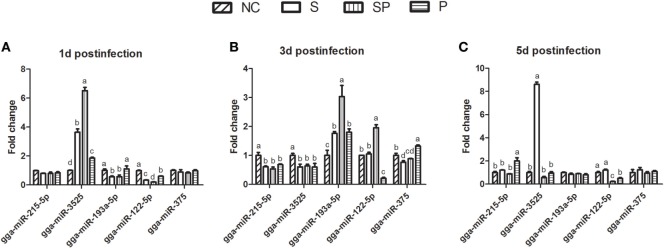
Expression of five randomly selected microRNA in ceca by qPCR. Different letters above bars indicate significant differences among treatments within each sampling day (*P* < 0.05). Samples were collected from one bird per cage (*n* = 6/treatment) at day 1 **(A)**, day 3 **(B)**, and day 5 **(C)** after oral gavage with PBS or *S*. Typhimurium. NC: an uninfected control group; S: a positive control group infected with *S*. Typhimurium; SP: a group infected with *S*. Typhimurium and orally supplemented with *Lactobacillus plantarum* Z01 (LPZ01); P: a group only orally supplemented with LPZ01.

To confirm and quantify the differential expression, five miRNAs (gga-miR-215-5p, gga-miR-3525, gga-miR-193a-5p, gga-miR-122-5p, and gga-miR-375) were randomly selected and validated by RT-PCR with six replicates of cecal samples in each group (Figure 3). The results indicated that the miRNA expression patterns were similar to those determined by the next-generation sequencing (NGS) (Figure S2 in Supplementary Material). One day after the *S*. Typhimurium infection, gga-miR-3525 increased (3.651, 6.508, 1.859 fold change in the S, SP, and P groups, respectively), while gga-miR-122-5p decreased in the S, SP, and P groups (0.667, 0.796, and 0.401 fold change, respectively) compared with the NC group. Gga-miR-193a-5p decreased in the S and SP groups (0.438- and 0.432-fold change, respectively) and was not significantly changed in the P group, while gga-miR-215-5p and gga-miR-375 were not significantly changed among different treatment groups. Three days post the challenge, expression of gga-miR-215-5p and gga-miR-3525 decreased significantly, while gga-miR-193a-5p increased in the S, SP, and P groups as compared with the NC group. The expression of gga-miR-122-5p increased 1.957-fold in the SP group and decreased 0.792-fold in the P group. Gga-miR-375 decreased in the S and SP group (0.231- and 0.115-fold change) and increased in the P group (1.324 fold change). At 5 days post *S*. Typhimurium challenge, expression of gga-miR-193a-5p and gga-miR-375 were not significantly changed in all treatment groups. Besides, gga-miR-215-5p increased in the P group (2.003 fold change) and gga-miR-3525 increased in the S group (8.604-fold change) compared with NC group. Gga-miR-122-5p decreased 0.782- and 0.501-fold change in the SP and P groups, respectively. Both the probiotic and *S*. Typhimurium changed the expression of miRNAs in the ceca of broilers.

In comparison with the S group, the SP group significantly increased gga-miR-3525 (1.783-fold change) expression on day 1 after the infection and gga-miR-193a-5p and gga-miR-122-5p expression on day 3 after the infection (1.719- and 1.860-fold changes, respectively). On the other hand, the SP group significantly reduced expression of gga-miR-3525 and gga-miR-122-5p on day 5 post the infection (15.227- and 5.509-fold changes, respectively). These results could reflect reduced numbers of *S*. Typhimurium and consequent less inflammation in ceca by the probiotics.

### Enriched KEGG Pathways and GO Functional Categories of Predicted Gene Targets of Differentially Expressed miRNAs

**Figure 4:**
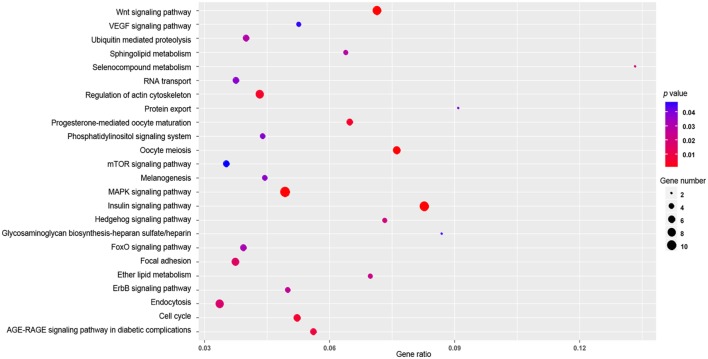
The enriched kyoto encyclopedia of genes and genomes (KEGG) pathways of target genes for 14 differentially expressed microRNAs. The *x*-axis indicates the gene ratio and the *y*-axis indicates the name of the KEGG pathway. The size of the dot indicates the number of target genes, and the color of the dot indicates different *p* value (Fisher’s Exact Test). The gene ratio indicates the ratio between the number of target genes associated with a KEGG pathway and the total number of genes in the pathway.

Target genes of 14 differentially expressed miRNAs (gga-miR-122-5p, gga-miR-193a-5p, gga-miR-194, gga-miR-215-5p, gga-miR-217-3p, gga-miR-375, gga-miR-1559-3p, gga-miR-1769-3p, gga-miR-1788-3p, gga-miR-1798-5p, gga-miR-2954, gga-miR-3525, and gga-miR-3531-3p) were predicted by using the miRDB and TargetScan programs. Three hundred forty-nine target genes of the 14 miRNAs were obtained by both miRDB and TargetScan programs (Table S2 in Supplementary Material). The enriched GO terms were listed in Table S3 in Supplementary Material, while enriched KEGG pathways were presented in Figure 4. Among the enriched pathways, more target genes were involved in the insulin signaling pathway, the MAPK signaling pathway and the Wnt-signaling pathway than in other pathways (Figure 4).

### Expression of Cytokine Genes

**Figure 5:**
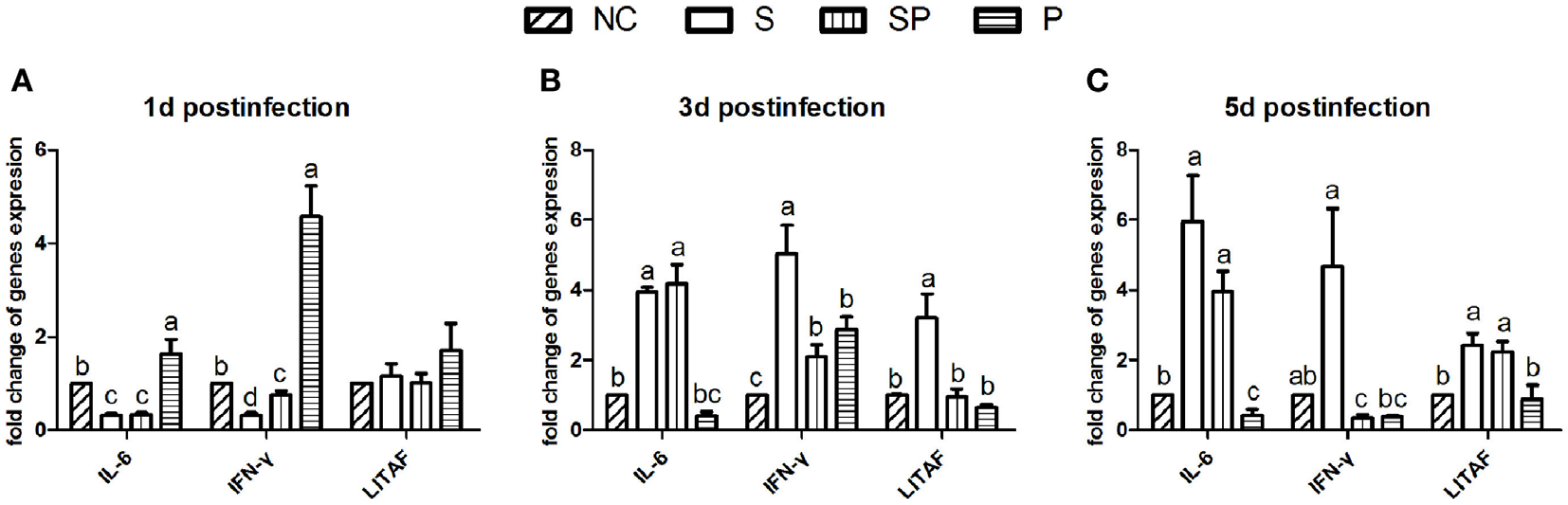
Relative expression of cytokines in ceca by qPCR. Expression of interleukin 6 (IL-6), interferon (IFN-γ), and lipopolysaccharide-induced tumor necrosis factor alpha factor (LITAF) at day 1 **(A)**, day 3 **(B)**, and day 5 **(C)** post the *S*. Typhimurium challenge was presented as the mean ± SD (*n* = 6). Different letters above bars indicate significant differences among treatments within each sampling day (*P* < 0.05). NC: an uninfected control group; S: a positive control group infected with *S*. Typhimurium; SP: a group infected with *S*. Typhimurium and orally supplemented with *Lactobacillus plantarum* Z01 (LPZ01); P: a group only orally supplemented with LPZ01.

The relative mRNA expression of interleukin (IL)-6, interferon (IFN)-γ, and lipopolysaccharide-induced tumor necrosis factor alpha factor (LITAF) over the course of the infection was evaluated using the qPCR method. One day post the *S*. Typhimurium challenge, the relative expression of IL-6 and IFN-γ was downregulated in the both S and SP groups and upregulated in the P group in comparison with the NC group, whereas no significant differences were observed in the expression of LITAF among treatment groups (Figure 5A). Three days after the infection, expression of IL-6 was significantly increased in the S and SP groups in comparison with the NC and P groups. At the same time point, the relative expression of LITAF and IFN-γ were significantly increased in the S group compared to those in other three groups (Figure 5B). Five days post the challenge, expression of IL-6, IFN-γ, and LITAF in the S group was higher than the other groups (Figure 5C).

In comparison with the S group, the SP group reduced expression of IFN-γ and LITAF on day 3 post the challenge and IFN-γ on day 5 post challenge. The results of these three pro-inflammation cytokines suggest that the LPZ01 contributed to decrease the inflammation of *S*. Typhimurium in the chicks.

### Relationship between miRNA Changes and Cytokine Changes

**Figure 6:**
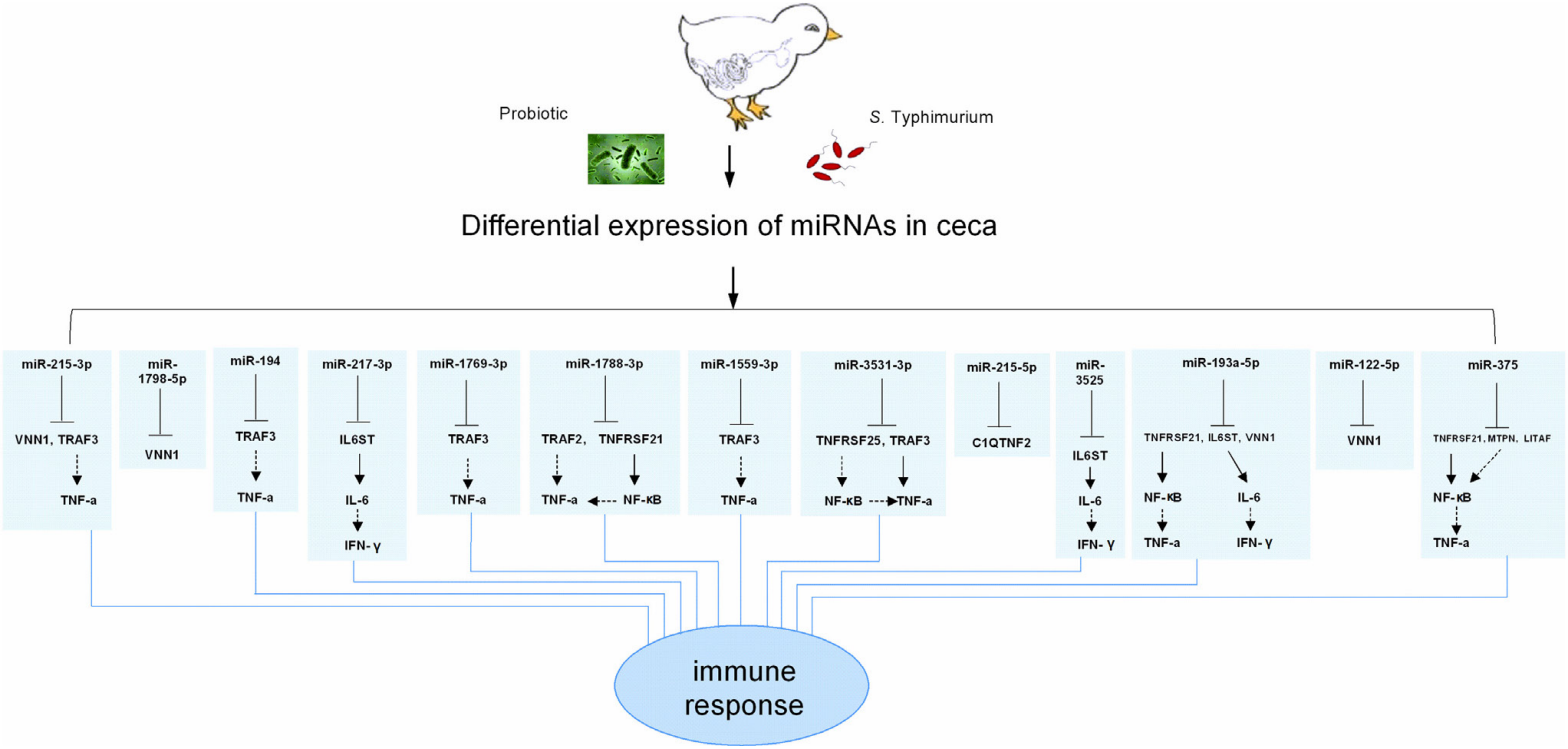
Overview of the known relationship between the differentially expressed microRNAs (miRNAs) and their targeted immune related genes. VNN1, TRAF3, TNF-α, IL6ST, IL-6, IFN-γ, TRAF2, TNFRSF21, TNFRSF25, NF-κB, C1QTNF2, MTPN, and LITAF are abbreviations of Vanin 1, tumor necrosis factor receptor associated factor 3, tumor necrosis factor-alpha, interleukin 6 signal transducer, interleukin 6, interferon gamma, tumor necrosis factor receptor associated factor 2, tumor necrosis factor receptor superfamily member 21, tumor necrosis factor receptor superfamily member 25, nuclear factor kappa B, C1q and tumor necrosis factor related protein 2, myotrophin, and lipopolysaccharide-induced tumor necrosis factor genes, respectively.

miR-1769-3p, miR-3531-3p, miR-1559-3p, miR-1798-5p, miR-1788-3p, miR-2954, and miR-3525 have not been found in the human and mouse genomes. On the other hand, miR-215-3p, miR-194, miR-217-3p, miR-215-3p, miR-193a-5p, miR-122-5p, and miR-375 are more conservative miRNAs in vertebrate animal species. Using the inflammation related target genes of differentially expressed miRNAs predicted by the TargetScan only, a potential relationship between miRNA changes and cytokine changes was presented in Figure 6. No immune related target genes were found for gga-miR-2954. Using vanin 1 (VNN1), LITAF, IFN-γ, and IL-6 as the main immune target genes, the remaining miRNAs were involved directly or indirectly in regulation of cytokine production, thus affecting the host immune responses to the bacteria infection. In particular, miR-3525 and miR-193a-5p were found to indirectly target IL-6 and IFN-γ.

## Discussion

Despite the recognized importance of miRNAs in host immune response to intestine inflammation in humans and mice, their comparative roles in regulating the immune response of birds are still not very clear. Expression surveys of chicken miRNAs have been reported for embryos (22), somites (23), lung, and trachea involved in immune responses induced by avian influenza virus infection (24) and spleen against Marke’s disease virus infection (25). This study was the first to interrogate miRNA expression changes in response to the *Salmonella* infection and the probiotic treatment in chickens.

The *Salmonella* challenge was carried out at the third day of age. During the first 3 days of life, chicken ceca were probably protected from incoming antigens by increased expression of gallinacin 1, 2, 4, and 6 (26). On day 4, peak expression of IL-8 and IL-17 has been observed that indicated minor and transient inflammation followed by normalization of the gut immune system and suppression of gallinacin expression (26). The NGS was used in this study to investigate the miRNA changes. The NGS approach is more suitable for small RNA discovery, which not only provides sequences of low abundance species but also provides quantitative data as the frequency of sequencing reads reflects the abundance of miRNAs in the population (27). In addition, many sequencing software tools have been developed to support miRNA data analysis from NGS data. In our study, we used the miRCat software to identify novel miRNAs. The identified 119 novel miRNAs still need to be validated by experiments (Table S1 in Supplementary Material). This experiment was carried out with *S*. Typhimurium. Other serotypes of *Salmonella* may induce different host responses and need to be further investigated.

Administration of probiotics to newly hatched chicks in this study protected chicks from *Salmonella* infection, as indicated by reduced bacterial counts in the SP group in comparison with that in the S group. The protective effects of probiotics could come from production of organic acids such as lactic acid and antimicrobial substances such as hydrogen peroxide, bacteriocins, and adhesion inhibitors as well as diminished access to the epithelial cells by the pathogenic bacteria used for the challenge (28). *S*. Typhimurium elicit the inflammation by its ability to penetrate the intestinal epithelium and to survive within macrophages (29). As a result, they alter host cell physiology, including production of pro-inflammatory cytokines. In the present study, *S*. Typhimurium challenge led to cecal inflammation. In comparison with the S group, expression of IFN-γ was decreased at 3 and 5 days post the infection, while expression of LITAF was decreased on d5 in the SP group (Figure 5). These results indicated that LPZ01 reduced *S*. Typhimurium*-*induced inflammation starting at 3 days after the challenge, since inflammation is characterized by the initial release of pro-inflammatory cytokines (INF-γ) (30) and tumor necrosis factor (TNF)-α (31) and they contribute to antimicrobial responses in the intestinal mucosa (30) and a breakdown in mucosal tolerance (31). TNF-α has yet not be identified in avian species and LITAF also known as TNF-α in chicken, which was upregulated following *in vitro* stimulation of macrophages for 4 h with *S*. Typhimurium endotoxin (32).

Target genes of 14 differentially expressed miRNAs in this study were predicted by the miRDB and TargetScan (Table S2 in Supplementary Material), since there is no specific program available for prediction of target genes of chicken miRNAs. Only those predicted by the both miRDB and TargetScan as target genes were selected for GO and KEGG analyses. The KEGG pathway analysis showed significant enrichments in the MAPK signaling and Wnt-signaling pathways, which were ranked among the most enriched pathways in the 24 KEGG pathways (Figure 4). Aballay et al. (33) found that *Caenorhabditis elegans* innate immune response triggered by *Salmonella enterica* was mediated by the MAPK Signaling Pathway. According to Ausubel (34), the innate immune response has ancient origins and conserved MAPK signaling cascades have common features of innate immunity in vertebrates and invertebrate animals. Similarly, GO analysis for this study also showed that the target genes were enriched significantly in negatively regulation of MAPK cascade, positive regulation of stress-activated MAPK cascade, negative regulation of stress-activated MAPK cascade, negative regulation of MAPK cascade, regulation of stress-activated MAPK cascade, stress-activated MAPK cascade, regulation of MAPK cascade, MAPK cascade (Table S3 in Supplementary Material). Therefore, our results support the notion that the MAPK signaling pathway is required for the host immune response triggered by *Salmonella* infection.

The Wnt signaling pathway is another one of the most enriched KEGG pathways in this study. Target genes of the 14 differentially expressed miRNAs were enriched in regulation of T cell activation, T cell aggregation, T cell activation, lymphocyte aggregation, negative regulation of T cell proliferation, regulation of activated T cell proliferation, activated T cell proliferation, alpha-beta T cell activation involved in immune response, T cell activation involved in immune response GO terms. In addition, the GO analysis also shows that target genes were significantly enriched for regulation of cAMP-dependent protein kinase activity, stress-activated MAPK cascade, immune system development, and regulation of immune system process (Table S3 in Supplementary Material). These results suggest significant roles of miRNAs in supporting the cecum in its growth and defense capabilities. The miRNA regulates target genes in the MAPK and Wnt signaling pathways, which in turn stimulates immune responses to the *S*. Typhimurium invasion.

Five miRNAs was selected and validated by RT-PCR (Figure 3). Their immune related target genes were predicted by the TargetScan only, since very few immune related target genes for the miRNAs were shared by both miRDB and TargetScan programs. In addition, published literature was searched for additional targeted genes. Gga-miR-215-5p was the most abundant miRNAs accounting for 40.54% of total reads of identified conserved miRNAs in 36 libraries. The abundance of miR-215-5p has previously been observed for the human intestinal-type epithelium by Bansal et al. (35). C1QTNF2 is a target gene of gga-miR-215-5p, which participates in the activation of MAPK activity.

Gga-miR-3525 was a unique miRNA to chickens and has been reported in the lung and tracheae of avian influenza virus infection chickens (24) and targets interleukin 6 signal transducer (IL6ST). IL6ST is a signal transducer of IL-6 (36), which is a multifunctional cytokine and involved in many immune related pathways, such as *Salmonella* infection and intestinal immune network for IgA production. In addition, IL-6 indirectly regulates the IFN-γ in the pathway of *Salmonella* infection.

The gga-miR-193a-5p targets tumor necrosis factor receptor superfamily member (TNFRSF) 21, which can regulate nuclear factor kappa B (NF-κB), a regulator of TNF-α. TNF-α is a member of a group of cytokines that stimulate the acute phase reaction in mammals. Although TNF-α has not been found nor described in the chicken genome, LITAF, which is the regulator for TNF-α expression in mammal (37), has been shown to play an important role in the intestinal inflammatory response in chickens (38). In addition, IL6ST and VNN1 are two target genes of has-miR-193a-5p. The polymorphisms of VNN1 gene is associated with susceptibility to inflammatory bowel diseases (39) and participates in the regulation of gut inflammation (40).

The gga-miR-122-5p targets the VNN1 in chickens (41). VNN1-knockout mice show decreased NSAID- and *Schistosoma*-induced intestinal inflammation and better controlled inflammatory reaction of intestine and intestinal injury (42). In addition to VNN1, human has-miR-122-5p targets IL6ST, interleukin 17 receptor (IL-17R), IL21R, tumor necrosis factor superfamily member 10 (TNFSF10), and tumor necrosis factor receptor superfamily member 1B (TNFRSF1B) genes to modulate the host reaction to bacteria and these target genes enriched in immune-related pathways, such as TNF signaling pathway, MAPK pathway. miR-375 is a key regulator of epithelial properties that are necessary for securing epithelium-immune system cross talk (43) and much less expression in the colons of patients with active ulcerative colitis (44). Myotrophin (MTPN), a previously validated target of miR-375 (45), which promotes dimerization of NF-κB subunits (46) and regulates NF-κB transcription factor activity. LITAF is another target gene of human has-miR-375.

The remaining 9 of 14 differentially expressed miRNAs excluding the above 5 miRNAs were also searched for their immune related target genes, using the same strategy. This exercise was performed in order to reveal the potential relationship between the differentially expressed miRNAs and cytokine changes. There was no immune related target gene for gga-miR-2954. As shown in the Figure 6, the remaining 13 differentially expressed miRNAs targeted cytokines LITAF, IFN-γ, and IL-6 directly or indirectly in response to the exogenous bacteria, both pathogenic *S*. Typhimurium and beneficial probiotics. In particular, miR-3525 and miR-193a-5p were found to indirectly target IL-6 and IFN-γ. The SP group significantly increased gga-miR-3525 expression on day 1 after the infection and gga-miR-193a-5p expression on day 3 after the infection (Figure 3), in comparison with the S group. This corresponded to the observed reduction of IFN-γ expression on day 3 and day 5 post the challenge by the SP group in comparison with the S group (Figure 5). Our results strongly suggested the linkage between expression of the miRNAs and cytokine production. Consequently, the host immunomodulation responses contributed to counteract the bacterial infection. Of course, how these miRNAs are actively involved in cytokine changes in the host needs further studies for confirmation. In particular, how different miRNAs work together deserves detailed studies.

To our knowledge, our study is the first comprehensive analysis of the miRNA expression patterns in ceca of broiler chickens. The target genes of the differentially expressed miRNAs were enriched in the MAPK signaling, Wnt-signaling, and insulin signaling pathways. The potential roles of miRNAs in regulation of cytokines and bacterial infection were explored. A total of 119 novel miRNAs were discovered in 36 libraries will most likely provide new insights into the contribution of the intestinal microbiota to understand the functions of host transcriptional landscape during infection by intracellular bacteria.

## Additional Information

All the raw sequencing data used in this work have been deposited in the NIH short read archive under accession number PRJNA309476.

## Ethics Statement

All experimental protocols used in this experiment were approved by the Northwest A&F University Institutional Animal Care and Use Committee (protocol number NWAFAC1025).

## Author Contributions

QLC and XZ contributed to the initial design of this experiment and prepared the manuscript of this publication. QLC, LXZ, and SYM performed the experiment. QLC, CT, and LLZ analyzed the data.

## Conflict of Interest Statement

The authors declare that the research was conducted in the absence of any commercial or financial relationships that could be construed as a potential conflict of interest.

## Footnotes

^1^http://hannonlab.cshl.edu/fastx_toolkit/commandline.html.

^2^http://srna-workbench.cmp.uea.ac.uk/tools/analysis-tools/mircat/.

^3^http://www.mirdb.org/miRDB.

## Supplementary Material

Figure S1 Length distribution and abundance of small RNAs. (A–C) The chicken ceca samples at 1, 3, and 5 days post *Salmonella* Typhimurium challenge, respectively.

Figure S2 The fold change trend analyses of differentially expressed microRNAs obtained by qPCR and next-general sequencing method. (**A–E**) The fold change trend of gga-miR-215-5p, gga-miR-3525, gga-miR-193a-5p, gga-miR-122-5p, and gga-miR-375, respectively. Sd1-NCd1: a positive control group infected with *Salmonella* Typhimurium compared to NC group 1 day after *S*. Typhimurium infection; SPd1-NCd1: a group infected with *S*. Typhimurium and orally supplemented with LPZ01 compared to NC group 1 day after *S*. Typhimurium infection; Pd1-NCd1: a group only orally supplemented with LPZ01 compared to NC group 1 day after *S*. Typhimurium infection; Sd3-NCd3: a positive control group infected with *S*. Typhimurium compared to NC group 3 days after *S*. Typhimurium infection; SPd3-NCd3: a group infected with *S*. Typhimurium and orally supplemented with LPZ01 compared to NC group 3 days after *S*. Typhimurium infection; Pd3-NCd3: a group only orally supplemented with LPZ01 compared to NC group 3 days after *S*. Typhimurium infection; Sd5-NCd5: a positive control group infected with *S*. Typhimurium compared to NC group 5 days after *S*. Typhimurium infection; SPd5-NCd5: a group infected with *S*. Typhimurium and orally supplemented with LPZ01 compared to NC group 5 days after *S*. Typhimurium infection; Pd5-NCd5: a group only orally supplemented with LPZ01 compared to NC group 5 days after *S*. Typhimurium infection.

Table S1 List of 119 novel microRNA identified for chickens.

Table S2 Predicted microRNA (miRNA) target genes of 14 differentially expressed miRNAs in chicken ceca.

Table S3 Gene ontology terms enriched by predicted target genes for 14 differentially expressed microRNA in chicken ceca.
