# Supplementary material for: Involvement of MicroRNAs in Probiotics-Induced Reduction of the Cecal Inflammation by Salmonella Typhimurium
Source: Front Immunol. 2017 Jun 13;8:704. doi: 10.3389/fimmu.2017.00704 (PMC5468434; doi:10.3389/fimmu.2017.00704)
Supplement: Figure S1 — Length distribution and abundance of small RNAs. (A–C) The chicken ceca samples at 1, 3, and 5 days post Salmonella Typhimurium challenge, respectively. [file Image_1.PDF]

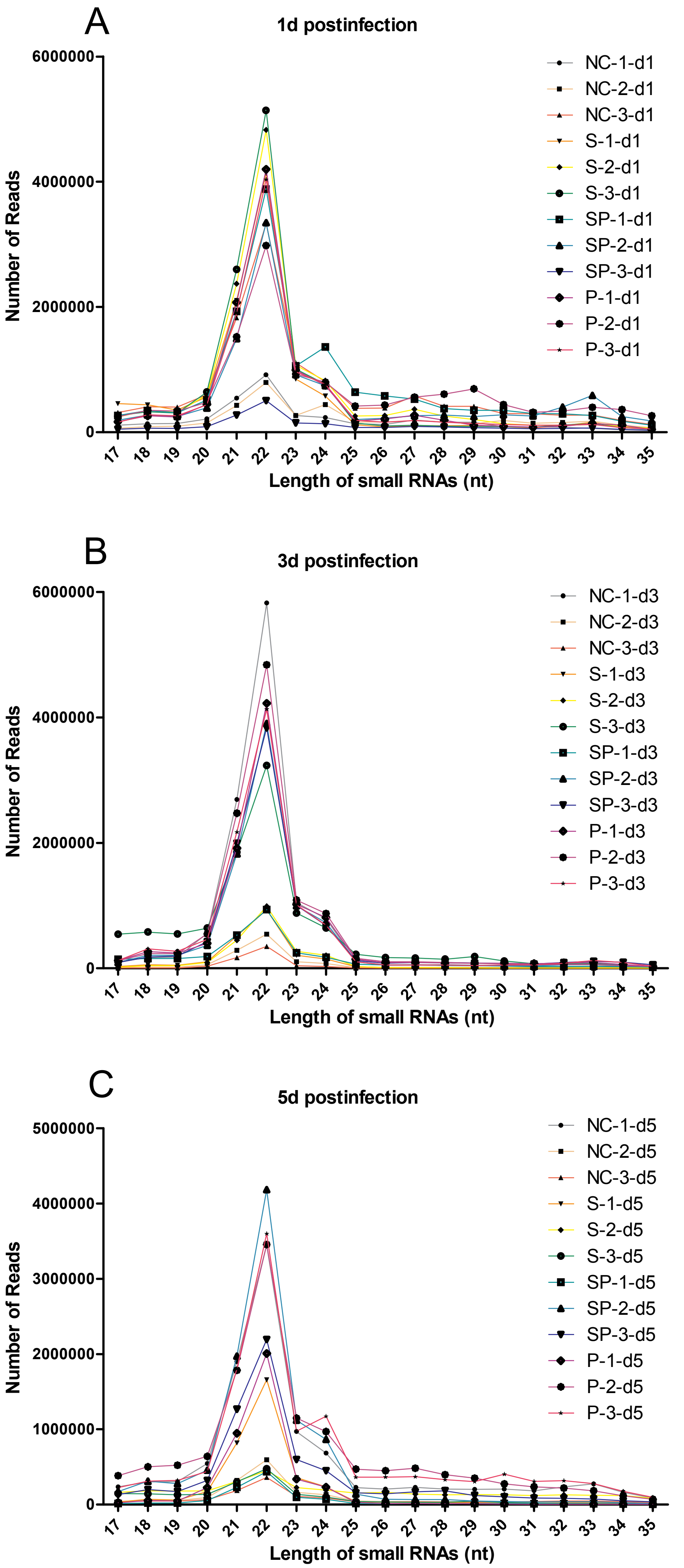
A
1d postinfection
Number of Reads
6000000
4000000
2000000
0
17 18 19 20 21 22 23 24 25 26 27 28 29 30 31 32 33 34 35
Length of small RNAs (nt)
NC-1-d1
NC-2-d1
NC-3-d1
S-1-d1
S-2-d1
S-3-d1
SP-1-d1
SP-2-d1
SP-3-d1
P-1-d1
P-2-d1
P-3-d1
B
3d postinfection
Number of Reads
6000000
4000000
2000000
0
17 18 19 20 21 22 23 24 25 26 27 28 29 30 31 32 33 34 35
Length of small RNAs (nt)
NC-1-d3
NC-2-d3
NC-3-d3
S-1-d3
S-2-d3
S-3-d3
SP-1-d3
SP-2-d3
SP-3-d3
P-1-d3
P-2-d3
P-3-d3
C
5d postinfection
Number of Reads
5000000
4000000
3000000
2000000
1000000
0
17 18 19 20 21 22 23 24 25 26 27 28 29 30 31 32 33 34 35
Length of small RNAs (nt)
NC-1-d5
NC-2-d5
NC-3-d5
S-1-d5
S-2-d5
S-3-d5
SP-1-d5
SP-2-d5
SP-3-d5
P-1-d5
P-2-d5
P-3-d5

Supplementary Figure 1. Length distribution and abundance of small RNAs. Panels **A, B, C** show the chicken ceca samples at 1d, 3d and 5d post *S*. Typhimurium challenge, respectively.
