# Supplementary material for: Involvement of MicroRNAs in Probiotics-Induced Reduction of the Cecal Inflammation by Salmonella Typhimurium
Source: Front Immunol. 2017 Jun 13;8:704. doi: 10.3389/fimmu.2017.00704 (PMC5468434; doi:10.3389/fimmu.2017.00704)
Supplement: Figure S2 — The fold change trend analyses of differentially expressed microRNAs obtained by qPCR and next-general sequencing method. (A–E) The fold change trend of gga-miR-215-5p, gga-miR-3525, gga-miR-193a-5p, gga-miR-122-5p, and gga-miR-375, respectively. Sd1-NCd1: a positive control group infected with Salmonella Typhimurium compared to NC group 1 day after S. Typhimurium infection; SPd1-NCd1: a group infected with S. Typhimurium and orally supplemented with LPZ01 compared to NC group 1 day after S. Typhimurium infection; Pd1-NCd1: a group only orally supplemented with LPZ01 compared to NC group 1 day after S. Typhimurium infection; Sd3-NCd3: a positive control group infected with S. Typhimurium compared to NC group 3 days after S. Typhimurium infection; SPd3-NCd3: a group infected with S. Typhimurium and orally supplemented with LPZ01 compared to NC group 3 days after S. Typhimurium infection; Pd3-NCd3: a group only orally supplemented with LPZ01 compared to NC group 3 days after S. Typhimurium infection; Sd5-NCd5: a positive control group infected with S. Typhimurium compared to NC group 5 days after S. Typhimurium infection; SPd5-NCd5: a group infected with S. Typhimurium and orally supplemented with LPZ01 compared to NC group 5 days after S. Typhimurium infection; Pd5-NCd5: a group only orally supplemented with LPZ01 compared to NC group 5 days after S. Typhimurium infection. [file Image_2.PDF]

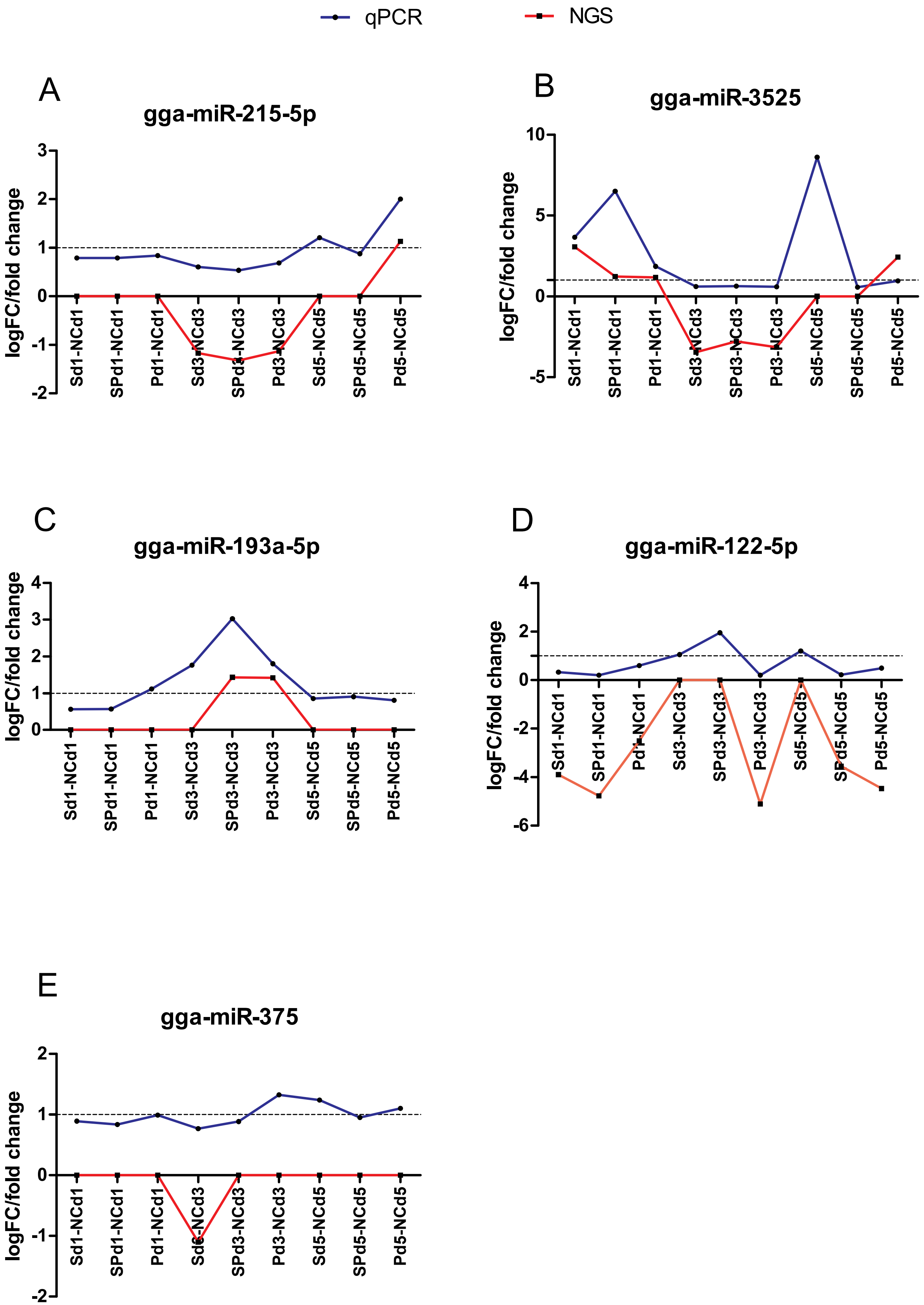
qPCR
NGS
A
gga-miR-215-5p
B
gga-miR-3525
C
gga-miR-193a-5p
D
gga-miR-122-5p
E
gga-miR-375
logFC/fold change
Sd1-NCd1
SPd1-NCd1
Pd1-NCd1
Sd3-NCd3
SPd3-NCd3
Pd3-NCd3
Sd5-NCd5
SPd5-NCd5
Pd5-NCd5

Supplementary Figure 2. The fold change trend analyses of differentially expressed miRNAs obtained by qPCR and next general sequencing (NGS) method. Panels **A, B, C, D** and **E** show the fold change trend of gga-miR-215-5p, gga-miR-3525, gga-miR-193a-5p, gga-miR-122-5p and gga-miR-375, respectively.

Sd1-NCd1: a positive control group infected with *S*. Typhimurium compared to NC group one day after *S*. Typhimurium infection; SPd1-NCd1: a group infected with *S*. Typhimurium and orally supplemented with LPZ01 compared to NC group one day after *S*. Typhimurium infection; Pd1-NCd1: a group only orally supplemented with LPZ01 compared to NC group one day after *S*. Typhimurium infection; Sd3-NCd3: a positive control group infected with *S*. Typhimurium compared to NC group three days after *S*. Typhimurium infection; SPd3-NCd3: a group infected with *S*. Typhimurium and orally supplemented with LPZ01 compared to NC group three days after *S*. Typhimurium infection; Pd3-NCd3: a group only orally supplemented with LPZ01 compared to NC group three days after *S*. Typhimurium infection; Sd5-NCd5: a positive control group infected with *S*. Typhimurium compared to NC group five days after *S*. Typhimurium infection; SPd5-NCd5: a group infected with *S*. Typhimurium and orally supplemented with LPZ01 compared to NC group five days after *S*. Typhimurium infection; Pd5-NCd5: a group only orally supplemented with LPZ01 compared to NC group five days after *S*. Typhimurium infection.
